# The association between iodine intake and semen quality among fertile men in China

**DOI:** 10.1186/s12889-020-08547-2

**Published:** 2020-04-06

**Authors:** Yu Sun, Chen Chen, Gordon G. Liu, Meijiao Wang, Cuige Shi, Ge Yu, Fang Lv, Ning Wang, Shucheng Zhang

**Affiliations:** 1 PKU China Center for Health Economic Research, Peking University, Beijing, 100871 China; 2 National School of Development, Peking University, Beijing, 100871 China; 3 National Research Institute for Family Planning, Beijing, 100081 China; 4 Affiliated Tumor Hospital of Harbin Medical University, Haerbin, 150040 China; 5 Clinical Medical College, Yangzhou University, Yangzhou, China; 6 Reproductive Medicine Center, Department of Obstetrical and Gynecology, Northern Jiangsu Peoples Hospital, Yangzhou, China

**Keywords:** Urinary iodine concentration, Semen quality, Semen counts, Semen motility, Time to pregnancy

## Abstract

**Background:**

Iodine intake is essential in the production of thyroid hormone but very few foods are rich in it. Iodine deficiency or excess iodine level may both lead to thyroid disorders, which further affects human fertility function. The objective of this study is to investigate the relationship between iodine intake and seminal parameters among fertile men in China.

**Methods:**

A total of 1098 couples were recruited by trained physicians at different family planning service stations in 2015. Semen and iodine samples were obtained from male respondents. A questionnaire survey inquired about demographic information from couples. The main outcome variables of semen quality were semen volume, semen concentration, semen motility, and sperm count, and time to pregnancy. Urinary iodine concentration (UIC) was used to measure iodine levels for male respondents. Ordinary least squared regressions and logistic regressions were performed to estimate the association between iodine intake level and semen quality parameters.

**Results:**

Male respondents with deficient or excess iodine levels had a 5% higher semen volume relative to those with optimal iodine intake (*p* < 0.1). Suboptimal iodine intake was negatively associated with semen concentration and semen counts (*p* < 0.01). Longer time of pregnancy was observed in iodine deficiency and excess group than those in the optimal group (*p* < 0.01).

**Conclusion:**

In general, iodine deficiency and excess were both associated with decreasing semen quality parameters in male respondents.

## Background

The needs of infertility care have increased remarkably over the past few decades [1–3]. Some treatment is expensive, such as assisted reproductive techniques, leading to a heavy economic burden on infertile couples [4–6]. Hence, there is an increasing interest in examining the factors associated with decreasing semen quality since semen quality is a significant determinant of male fertility and is taken as a clinical measurement of male fecundity. Semen quality is frequently assessed using the WHO criteria by the following parameters: semen volume, semen count, and semen motility (including progressive sperm, nonprogressive sperm, and immotile sperm) [7]. Past literature indicates that risk factors include genetic factors, congenital and intrauterine factors such as age, smoking, alcohol uses, environmental factors, endocrine, and nutrition intake [8]. Iodine intake, a factor that is closely related to endocrine and nutrition intake, has drawn little attention in its association with male reproduction function.

Very few foods are rich in iodine, including iodized salt, milk, and seaweed, etc. [9, 10]. Nevertheless, iodine is essential in the production of thyroid hormones, which is responsible for regulating enzymes and organic processes that are necessary for life [11]. Iodine deficiency or excess iodine level may both lead to thyroid disorders, which further affects human fertility function. Partal-Lorente et al. find higher iodine levels are associated with lower motile sperm counts and more than 3 years of not achieving pregnancy among males [12]. Another research suggests that excessive iodine intake may be a potential cause for discontinuous decline in sperm counts in the United States, France, and the United Kingdom [13]. Most of the literature exploring the relationship between iodine intake and semen quality are carried out in Western countries and little is known about the association between iodine intake and male fertility in China.

The universal salt iodization programme in 1994 has been a globally recognized story for its achievement in preventing iodine deficiency in China. However, challenges of the coexistence of iodine deficiency and excess have been observed in China from the last decade [14, 15]. As the consumption of processed foods has been increasing, there is a declining trend of iodine intake because of the reductions in iodized salt consumption. This declining trend is also due to people’s concern about excess iodine intake and thyroid disease. On the contrary, there are areas with high iodine content in the water, leading to the risk of excess iodine intake for the local residents [16].

A recent study shows that the semen quality of Chinese men has been decreasing over the past decades [17]. Therefore, it is important to explore the factors associated with semen quality, especially the effects of iodine intake. This study focused on investigating the physiology of fertile male reproduction function and selected males with pregnant partners to rule out underlying infertility factors relevant to females. Exploring the causes of female infertility is not the objective of our project. The results of this study could provide vital implications for both nutrition and reproduction health policies. Whether and how iodine intake may affect male reproduction function remains unclear and it is crucial for future guidelines for public nutrition education and policy initiatives. In addition, the information may provide some suggestions for maintaining individual reproduction health.

## Methods

### Study population

The current study involved heterosexual couples with female partners being pregnant within the past 3 months because semen analyses within 3 months were more representative of the males’ fertility. The couples were recruited from family planning service institutions by trained staff from June to November in 2015. The participants were from seven provinces: Heilongjiang, Henan, Hebei, Jiangxi, Zhejiang, Guangxi, and Xinjiang. They needed to fill a form to provide a history of the disease and drug use for preliminary screening. The couples should be aged 18 years old and over and be permanent local-residents. The pregnancy should be naturally conceived. The couples were excluded if the male partner had one of the following conditions: being exposed to iodine products except for edible iodine within past 2 weeks; being diagnosed with chronic diseases; being diagnosed of thyroid-related disease, such as hyperthyroidism, hypothyroidism, and immune thyroid disease; injuries of reproduction system and surgeries performed in the genital area; being diagnosed of sexually transmitted diseases; urogenital disease (e.g., male urogenital infections, cryptorchidism and varicocele); working associated with potential occupational hazards (e.g., petrochemical industry and occupational environment of high temperature and humidity); having records of high-risk reproductive toxicant (volatile solvents like benzene/gasoline diesel/aldehyde alcohol and radiological rays such as radiation/electromagnetic/radar/microwave). All analyses of semen and urine samples were performed by trained technicians in close laboratories.

To determine the sample size for this study, we utilized the following formula to calculate the sample size:

$$ N=D\ast p\ast \left(1-p\right)\ast {\left(\frac{Z_a}{d}\right)}^2 $$

The margin of error was set to be 5% and the confidence level is 95%. We specified 50% as sample proportion and D equaled 1.5 reflecting the efficiency of study design. At least 256 respondents should be included to achieve validity. In fact, we recruited a total of 1277 couples in this study. After removing couples who met exclusion criteria, 1098 couples were included in the final analyses. Male respondents and their partners were excluded from analyses due to the following reasons: 39 men had sexual abstinence less than 2 days, 13 male respondents were diagnosed with chronic diseases, 94 men had thyroid-related diseases, 13 men were excluded due to urogenital diseases or surgeries, and 20 male respondents were identified with occupational hazards.

All participants were informed about this study and signed consent forms before completing the questionnaire, agreeing to donate the sample of semen and a random sample of midstream specimen of urine for the use in the current scientific research. Each male was economically compensated with an amount of 200 Chinese Yuan as compensation for travel. The present study was approved by the Ethics Committee of National Research Institute for Family Planning.

### Semen parameters measurement

Each participant provided a semen sample by masturbation into a plastic sterile container in a private room near the laboratory. Semen plasma was obtained after 2–7 days of sexual abstinence. After complete liquefaction, all semen samples were examined by physicians following recommendations from the “World Health Organization (WHO) laboratory manual for the examination and processing of human semen” (Fifth edition) [7]. Outcomes for sperm analyses included semen volume, semen count, and semen motility (categorized into progressive sperm, nonprogressive sperm, and immotile sperm). According to the WHO Laboratory Manual, semen volume was measured by weighing the sample in the vessel in which it was collected and sperm concentration was assessed by Neubauer hemocytometer. Total sperm count was obtained by multiplying the sperm volume with the sperm concentration. Sperm motility was measured by the average of two recordings from two independent physicians who analyzed sperm smears.

### Urinary iodine measurement

We utilized urinary iodine concentration (UIC) to measure iodine levels because it was the most common indicator of iodine nutrition [18]. Participants provided a random sample of midstream specimen of urine on the spot. The assessment of UIC was performed by the Sandell-Kolthoff method. This method was a widely-used method due to its low cost and simple detection of iodine via spectrophotometric analysis. We divided the sample into three groups based upon the standards proposed by WHO/UNICEF/ICCIDD: iodine deficient group, UIC < 100 μg/L; iodine optimal group, 100 ≤ UIC < 200 μg/L; iodine excess group, UIC ≥ 200 μg/L) [19, 20].

### Other control variables

Demographic information was obtained from a questionnaire before the semen plasma and urine samples were collected. The questionnaire inquired about the information regarding age, education, occupations, health behaviors and Hukou status. Hukou status was assigned to each household to classify the residents by their place of birth. Each type of Hukou was entitled to different rights, so it was a variable showing the socioeconomic status of the respondents. Hukou status was divided into three categories in the questionnaire, including urban, rural and suburban Hukou status. Anthropometric data for height and weight were collected by registered physicians. Health behaviors were measured by smoking status and drinking status. The questionnaire also included questions about sexual abstinence duration, times of pregnancy and number of living birth. In addition, the female partners of the participants provided the time to pregnancy (TTP) with help from registered physicians. TTP was defined as the number of months from stopping contraception to getting pregnant and was utilized as a standardized measurement of fertility [21]. GDP per capita of each province was collected from the 2015 Statistical Yearbook from the National Bureau of Statistics of China. Information about PM 2.5 was collected from a report of “Ranking of PM2.5 concentration in 366 cities in China” published by Greenpeace.

### Statistical analysis

The descriptive statistics were presented as means for continuous variables and percentages for categorical variables with standard deviations. The main outcome variables of semen quality were semen volume, semen concentration, semen motility, and sperm count, and time to pregnancy. Because semen volume, semen count, and semen concentration followed skewed distributions, the natural log-transformed outcomes were applied in the regression. Ordinary least squared regressions were performed to estimate the association between iodine intake level and the outcomes, including semen volume, semen concentration, and semen count and time to pregnancy. For the outcome of semen quality, we divided the male participants into two groups as sperm motility being either low (below 40%) or high (greater or equal 40%) and utilized logistic regression models [16]. Covariates included age, body mass index (BMI), education level (illiterate, primary school, high school, and college and above), indicators of smoking (smoking vs. non-smoking), frequencies of alcohol consumption (none, occasional, and often), sexual abstinence time, GDP per capita and indicator of poor air quality. Per capita GDP was included in the model as a proxy of income. Existing literature suggested that income level affected dietary knowledge and nutrition intake among Chinese population, which would further affect individual’s iodine intake [22]. Exposure of PM2.5 was also controlled in the regression since evidence showed that PM2.5 was associated with male reproductive disorders [20]. Sensitivity analysis was performed afterwards to ensure the robustness of the results. In the sensitivity analysis, regression methods and variables were identical to the main methods, but sample were restricted to those with the time to pregnancy up to and including 12 months. Statistical analyses were carried out using Stata 14.0 version [23].

## Results

### Participant characteristics

Table 1 presented the characteristics of male respondents of the whole sample and by iodine concentration levels. Participants were all married with a mean age of about 27 years old across different iodine concentration groups. More than half of the study population were graduates from middle schools or high schools (67%) and about half of the respondents (48%) were from rural areas. Both the lowest proportion of smoking (32%) and the highest proportion of never-drinking (52%) were observed in the group of optimal iodine levels. The average sexual abstinence duration lasted about 4 days. Overall, there were no significant differences in most of the characteristics in comparisons of deficiency vs. optimal and excess vs. optimal.

**Table 1:** Summary of characteristics of study male participants (means ± SD, or % ± SD) by iodine status groups

Characteristic	Whole Sample (*n* = 1098)	Iodine deficiency group (*n* = 275)	Iodine optimal group (*n* = 407)	Iodine excess group (*n* = 416)
Age (years)	27.47 (3.85)	27.42 (3.79)	27.54 (3.98)	27.42 (3.75)
BMI (kg/m2)	23.02 (2.58)	23.1 (2.67)	22.95 (2.63)	23.04 (2.47)
Education				
Primary school and less (%)	3(0.17)	1(0.09)	2(0.16)	5(0.21)
Middle and high school (%)	67(0.47)	68(0.47)	64(0.48)	71(0.46)
College and above (%)	30(0.46)	31(0.46)	34(0.47)	25(0.43)
Hukou status				
Urban (%)	38(0.49)	33(0.47)	36(0.48)	43(0.50)
Rural (%)	48(0.50)	48(0.50)	49(0.50)	48(0.50)
Suburban (%)	14(0.35)	19(0.4)	14(0.35)	10(0.30)
Smoking (%)	36(0.48)	39(0.49)	32(0.47)	37(0.48)
Drinking habits				
Never-drinking (%)	47(0.50)	40(0.49)	52(0.50)	45(0.5)
Occasional-drinking (%)	7(0.25)	8(0.28)	6(0.24)	7(0.25)
Often-drinking (%)	47 (0.50)	52(0.50)	42(0.49)	48(0.5)
Abstinence duration (days)	4.12 (1.52)	4.08(1.52)	4.12(1.51)	4.15(1.54)

*BMI* body mass index. Iodine deficient group, UIC < 100 μg/L; iodine optimal group, 100 ≤ UIC < 200 μg/L; iodine excess group, UIC ≥ 200 μg/L

### Summary of urine and semen parameters

Table 2 displayed a summary of urine iodine and semen parameters for study participants. WHO 2010 standards were displayed in the table for comparison [7]. In addition, we use the optimal group as the reference group to compare it with the other two sub-groups for each semen parameter. The semen parameters displayed significant differences in semen volume and semen count when comparing those parameters of deficient or excess group to optimal group. The mean of the semen volume of the iodine deficiency group and the excess group was higher than the average volume of iodine optimal group. While the semen count was much higher in the optimal group relative to the other groups. We observed no significant differences in sperm motility measurements across different groups. Table 2 showed that the semen volumes and percentage of nonprogressive sperm were higher in our sample than the WHO standards.

**Table 2:** Summary of semen biochemical variables for study respondents

	2010 WHO Standards^a^	Whole sample (*n* = 1098)	Iodine deficiency group (*n* = 275)	Iodine optimal group (*n* = 407)	Iodine excess group (*n* = 416)
Semen volume (mL)	1.5	3.02 (2.94, 3.09)	3.09^**^ (2.94, 3.25)	2.92 (2.80, 3.03)	3.06^**^ (2.94, 3.18)
Semen concentration (million/ml)	15	58.23 (−10.89, 127.36)	41.21^***^ (−10.48, 92.91)	70.86 (− 68.49, 210.21)	57.13^***^ (− 44.03, 158.29)
Semen count (million)	39	175.98 (− 791.93, 1143,90)	128.68 (− 731.69, 989.05)	208.92 (− 1866.47, 2284.32)	175.02 (− 1274.32, 1624.37)
Semen motility (%)	40	51.33 (42.63, 60.03)	51.75 (35.04, 68.47)	51.61 (36.61, 66.60)	50.78 (36.87, 64.68)
Progressive sperm (%)	32	43.85 (34.12, 53.59)	44.08 (24.87, 63.28)	44.16 (27.88, 60.44)	43.41 (27.60, 59.22)
Nonprogressive sperm (%)	1	7.48 (5.97, 8.98)	7.68 (4.58, 10.79)	7.45 (5.18, 9.71)	7.37 (4.75, 10.00)

*UIC* urinary iodine concentration. Group comparisons are performed by t-test for continuous variables and chi-squared tests for categorial variables using iodine optimal group as the reference group

^a^2010 World Health Organization (WHO) recommendations for semen parameters

***, statistically significant at the 1% level; ** statistically significant at the 5% level; * statistically significant at the 10% level

**Fig. 1:**
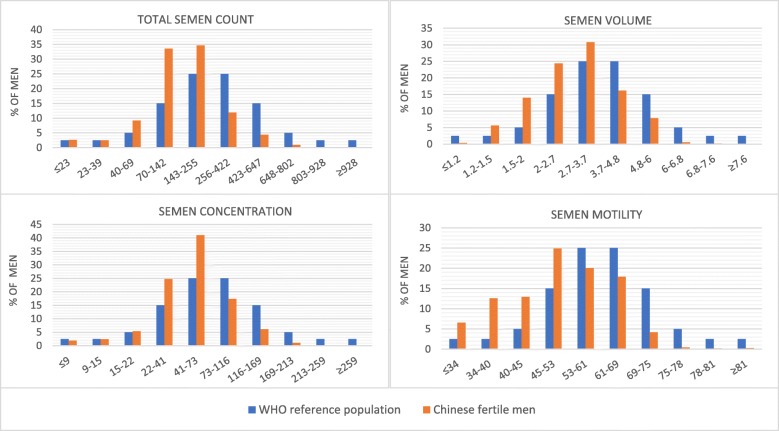
Distribution of semen parameters in Chinese fertile men (orange bars) compared to the WHO reference population (blue bars). Data of Chinese fertile men is from our sample, data of the WHO reference population is from a study conducted by Cooper et al. (2010). The study population for the WHO reference group were fertile men (fathers with TTP ≤ 12 months) in eight countries on three continents. See “Study Population” and “Summary of Urine and Semen Parameters” for details

Figure 1 showed the distributions of different semen parameters among Chinese men (orange bars) in this study compared to the distribution of the WHO reference group (blue bars) [24].The study population for the WHO reference group were fertile men (fathers with TTP ≤ 12 months) in eight countries on three continents. The categories in the reference levels were defined using distributions of semen parameters from fertile men with a TTP of 12 months or less. The upper left panel in Fig. 1 indicated that more men were concentrated in the left part of distribution than reference group. According to the upper right panel, semen volume among Chinese men were concentrated in the lower and middle interval of distribution compared to the reference group. The lower left panel manifested that more men were concentrated in the middle distribution of semen concentration relative to the reference group. Sperm were more likely to be immotile for Chinese men according to the lower right panel.

### Potential effects of iodine intake on semen parameters

Regression results of semen quality parameters on unrainy iodine level and other control variables were demonstrated in Table 3. After controlling for health behaviors, demographics, occupation characteristics, and Hukou status, the coefficients of unrainy levels were significant at the 0.1 to 0.01 level across 4 semen parameters except semen motility.

**Table 3:** Regression coefficients for key variables ^a^

	(1)	(2)	(3)	(4)	(5)
	Semen Volume ^b^	Semen concentration ^b^	Semen count ^b^	Semen Motility ^c^	Time to pregnancy
**Unrainy iodine level: reference group (iodine optimal)**					
Iodine deficiency	0.05^*^ (0.03)	−0.58^***^ (0.05)	− 0.52^***^ (0.06)	0.02 (0.03)	3.49^***^ (0.39)
Iodine Excess	0.06^*^ (0.03)	−0.26^***^ (0.04)	−0.20^***^ (0.05)	− 0.02 (0.02)	1.42^***^ (0.28)
**BMI category: reference group (normal weight)**					
underweight	−0.01 (0.06)	0.05 (0.10)	0.04 (0.13)	−0.14^**^ (0.06)	− 0.54 (0.73)
Overweight	0.01 (0.03)	−0.04 (0.05)	−0.02 (0.13)	0.01 (0.03)	0.07 (0.36)
Obese	0.02 (0.10)	0.40^**^ (0.16)	0.42^**^ (0.26)	−0.08 (0.09)	3.52^***^ (1.21)
**Drink frequency: reference group (never drink)**					
Occasional drink	0.01 (0.05)	−0.02 (0.08)	−0.00 (0.10)	− 0.07 (0.05)	−0.77 (0.73)
Often drink	0.01 (0.02)	0.11^***^ (0.04)	0.13^***^ (0.05)	−0.03 (0.02)	0.11 (0.29)
Smoking	−0.01 (0.02)	−0.03 (0.04)	− 0.04 (0.05)	0.01 (0.02)	1.05^***^ (0.31)
Sexual abstinence duration	0.02^**^ (0.01)	0.04^***^ (0.01)	0.06^***^ (0.01)	0.00 (0.01)	−0.19^**^ (0.09)
Age	0.00 (0.00)	0.01^**^ (0.00)	0.02^***^ (0.01)	−0.00 (0.00)	0.04 (0.04)
Per capita GDP	0.00^*^ (0.00)	−0.00 (0.00)	0.00 (0.00)	−0.00^**^ (0.00)	0.00 (0.00)
Poor air quality	0.04 (0.03)	0.09^**^ (0.04)	0.13^***^ (0.05)	−0.07^***^ (0.02)	1.19^***^ (0.30)
Observations	1098	1098	1098	1098	1098

^a^Other controlled variables include: education (primary school and less, middle and high school, college and above), occupation (heavy labor vs. other), and Hukou status (urban, rural and suburban)

^b^Semen volume, semen concentration and semen count were natural-log transformed

^c^Semen motility was a dummy indicator, with 1 indicating greater than 40%

^*^p < 0.1, ^**^p < 0.05, ^***^p < 0.01; 2. Standard errors in parentheses

Compared to men with optimal iodine levels, men with deficient or excess iodine levels had 5% higher semen volume in the collected sample (Column [1]). Both iodine deficiency or iodine excess were associated with lower semen concentration (58% and 26% respectively), indicating lower semen quality (Column [2]). Similarly, 52% and 20% fewer semen counts were observed in male respondents with iodine deficiency or iodine excess compared to those with optimal iodine levels (Column [3]). We did not observe a statistically significant association between iodine level and semen motility according to the results from Column [4]. Longer duration of time to pregnancy was found in male respondents with deficient or excess level of iodine compared to those in optimal group (3.54 and 1.48 more months to pregnancy of enrolled couples respectively).

### Potential effects of other factors

Per capita GDP was an indicator of economic wellbeing and was positively associated with the sperm volume and negatively associated with the likelihood of having low semen motility. However, the association between per capita GDP and semen quality variables were either quite small or not significant. PM 2.5 was a measurement of environmental impacts and was significantly associated with all sperm parameters except sperm volume. Specifically, being exposed to poor air quality was positively associated with sperm volume and sperm concentration (an increment of 9% and 13% respectively). Higher air pollution was correlated with longer TTP and lower motile sperm.

Compared with being normal weight, being underweight was negatively associated with semen motility. In addition, being obese was positively correlated to semen concentration, semen count. Moreover, being obese increased a couple’s time to pregnancy by 3.52 months.

Compared with male respondents who never drink, those who often drink had 12% higher semen concentration and 13% more semen count. The drinking pattern was not related to other semen quality parameters and time to pregnancy.

Smoking was associated with longer time to pregnancy. Compared with non-smokers, being a smoker increase a couple’s time to pregnancy by 0.99 months. Smoking status was not associated with semen volume, semen concentration, semen count and semen motility.

Sexual abstinence duration was another variable that was significantly correlated to higher semen quality. Within 2–7 days of sexual abstinence duration, 1 more day of sexual abstinence duration increased semen volume by 2%, increased semen concentration by 3%, increased semen count by 4%.

### Sensitivity analysis

In sensitivity analysis, regression analyses were restricted to male respondents with TTP up to and including 12 months since infertility was usually defined as a failure of pregnancy within 12 months of unprotected intercourse [25, 26]. Most of the results remained similar and robust, except the effects of iodine intake on semen volume became no longer significant.

## Discussion

Improper iodine intake has been shown to impair male reproduction function in animals, such as adult male rats [27, 28] while the evidence for this association among males is scant. Iodine nutrition intake has been greatly improved after salt iodization since 1994 in China and different regions manifested different risks for iodine deficiency or excess. In this study, we utilized a cross-sectional design to investigate the potential association between iodine intakes and male reproduction function parameters, which to our knowledge is the first trial using the Chinese population. In general, the lower or higher urinary iodine level relative to the normal level was associated with lower sperm quality parameters. Furthermore, we observed that iodine deficiency might exert more adverse influences on semen quality parameters than iodine excess from the magnitudes of regression coefficients.

Male with optimal iodine intake was observed with lower semen volume, higher semen concentration, and greater semen counts. The mechanisms underlying the relationship between iodine intakes and semen quality might be through thyroid hormones. In general, thyroid hormones affect male reproductive function by altering serum testosterone levels and regulating seminal plasma components like calcium, fructose, magnesium, zinc, etc. Optimal testosterone level improves spermatogenesis and sperms count. Testosterone together with the seminal components affects indicators for semen quality, such as sperm motility and morphology [29]. Since thyroid disorders are often the consequence of iodine deficiency or excess iodine intake, the role of iodine intake that plays in male reproduction function could be important. However, we exclude male respondents with a diagnosis of thyroid-related diseases in the current study and could not examine the role of thyroid hormones in the relationship between iodine intake and semen quality.

Though iodine intake above or below the optimal level of iodine nutrition is both harmful to semen concentration and semen count, iodine deficiency could be more harmful. While iodine deficiency has been significantly decreased after salt iodization, several regions are still iodine deficient [14]. Iodine supplementation could be encouraged in areas with low iodized salt coverage as increased iodine intake could help reduce the improve semen quality.

The results also indicate that male respondents with optimal iodine intake tend to have shorter TTP relative to those with insufficient or excessive iodine intake. TTP has been widely used in reproductive epidemiology and a failure of being pregnant during a 12-month TTP is considered as infertility in clinical practice. However, TTP is a sensitive outcome and is susceptible to unobserved confounding factors [30]. Though the male respondents in our study are fertile men whose partners are pregnant at the time of data collection, this finding could not enable us to conclude that increasing male iodine intake to an optimal level would decrease TTP.

Our findings were consistent with previous studies of different samples and measurements of semen quality. A recent study was conducted in Spain and utilized both urinary and semen iodine to investigate the relation between iodine intake and semen quality among infertile males [12]. The results of this study showed that lower motile sperm count was found among men with higher urinary iodine levels. A higher urinary iodine level was found among men who failed to achieve pregnancy within 3 years than those being pregnant within3 years. A decrease in sperm count was found in the United States, France, and the United Kingdom after the introduction of iodine supplements in those areas [13]. Several studies found that higher PM2.5 was found to be negatively associated with sperm motility, which was similar to our results for semen motility [31, 32].

This study included a relatively large sample of fertile couples from ten counties in seven provinces in China. The results provided essential implications for reproduction health for adult couples. However, there were several limitations could not be addressed in this study. Firstly, our respondents were recruited in 2015 and constituted a cross-sectional sample, thus we could not establish a causal inference of the relationship between iodine intake and semen quality. Secondly, we focused on male respondents with proven fertility and did not include infertile men as comparison groups, leading to an underestimation of the association between iodine intake and semen quality. The present study was designed to focus on fertile couples because it would exclude the female-related infertile problems and provided a clean association between two factors in male respondents. In addition, it was possible that the influence of iodine nutrition on semen parameters might exhibit different patterns among infertile couples due to unobserved factors, such as genetic factors. However, proven fertility men were the most relevant reference group in studies of infertility. The information in this study might be useful for those with the diagnosis of infertility. Thirdly, information on other variables (like detailed smoking status and nutrients) that affect semen quality parameters were not available for our study. For example, details of the smoking status (e.g. number of cigarettes per day and smoking history) were not collected in the questionnaire. Therefore, the coefficient of smoking status in this study may not reveal the association of different smoking status in the real world and semen quality.

## Conclusions

In sum, the results of this study indicate that iodine deficiency and excess are both associated with decreasing semen quality parameters in Chinese fertile men compared to optimal iodine intake. Further studies of the general population, including both fertile and infertile men, will be required to examine the relationship between iodine intake and semen quality in China. The underlying mechanisms of this association should be thoroughly studied to provide effective methods of promoting fertility and decreasing infertility.

## Data Availability

The datasets generated and/or analyzed during the current study are not publicly available due to confidentiality agreement but are available from the corresponding author on reasonable request.

## Abbreviations

TTP: Time to pregnancy
WHO: World Health Organization
UIC: Urinary iodine concentration
BMI: Body mass index

## References

[1] Priti S, Schimenti JC (2015). The genetics of human infertility by functional interrogation of SNPs in mice. Proc Natl Acad Sci U S A.

[2] Rao N, Esber A, Turner A, Mopiwa G, Banda J, Norris A (2018). Infertility and self-rated health among Malawian women. Women Health.

[3] Temidayo S, Stefan S (2018). Diabetes mellitus and male infertility. Asian Pac J Reprod.

[4] Howard S (2018). The hidden costs of infertility treatment. BMJ.

[5] Group ECW (2015). Economic aspects of infertility care: a challenge for researchers and clinicians. Hum Reprod.

[6] Copel J (2014). Economic analysis of use of pessary to prevent preterm birth in women with multiple pregnancy (ProTWIN trial). Ultrasound Obstet Gynecol.

[7] World Health Organization DoRHaR (2010). WHO laboratory manual for the examination and processing of human semen.

[8] Halling J, Petersen MS, Jørgensen N, Jensen TK, Grandjean P, Weihe P (2013). Semen quality and reproductive hormones in Faroese men: a cross-sectional population-based study of 481 men. BMJ Open.

[9] Franke K, Meyer U, Wagner H, Flachowsky G (2009). Influence of various iodine supplementation levels and two different iodine species on the iodine content of the milk of cows fed rapeseed meal or distillers dried grains with solubles as the protein source. J Dairy Sci.

[10] Truong T, Baron-Dubourdieu D, Rougier Y, Guénel P (2010). Role of dietary iodine and cruciferous vegetables in thyroid cancer: a countrywide case–control study in New Caledonia. Cancer Causes Control.

[11] Zimmermann MB, Boelaert K (2015). Iodine deficiency and thyroid disorders. Lancet Diabetes Endocrinol.

[12] Partal-Lorente AB, Maldonado-Ezequiel V, Martinez-Navarro L, Herrera-Contreras I, Gutierrez-Repiso C, Garcã­A-Fuentes E, et al. Iodine is associated to semen quality in men who undergo consultations for infertility Reprod Toxicol 2017;73:1–7. 10.1016/j.reprotox.2017.07.020 28755858

[13] Sakamoto KQ, Ishizuka M, Kazusaka A, Fujita S (2004). Iodine intake as a possible cause of discontinuous decline in sperm counts: a re-evaluation of historical and geographic variation in semen quality. Jpn J Vet Res.

[14] Yang D, Yanhui G, Fangang M, Shoujun L, Zhipeng F, Junhua W (2014). Iodine deficiency and excess coexist in China and induce thyroid dysfunction and disease: a cross-sectional study. PLoS One.

[15] Sun D, Codling K, Chang S, Zhang S, Shen H, Su X, et al. Eliminating Iodine Deficiency in China: Achievements, Challenges and Global Implications. Nutrients. 2017;9(4):361. 10.3390/nu9040361 PMC5409700 28379180

[16] Cui SL, Liu P, Su XH, Liu SJ (2017). Surveys in areas of high risk of iodine deficiency and iodine excess in China, 2012-2014: current status and examination of the relationship between urinary iodine concentration and goiter prevalence in children aged 8–10 years. Biomed Environ Sci.

[17] Wang L, Zhang L, Song XH, Zhang HB, Xu CY, Chen ZJ (2017). Decline of semen quality among Chinese sperm bank donors within 7 years (2008-2014). Asian J Androl.

[18] World Health Organization (2007). Assessment of iodine deficiency disorders and monitoring their elimination: a guide for programme managers.

[19] World Health Organization (1999). Progress towards the elimination of iodine deficiency disorders (IDD).

[20] Zhou L, Su X, Li B, Chu C, Sun H, Zhang N (2019). PM2.5 exposure impairs sperm quality through testicular damage dependent on NALP3 inflammasome and miR-183/96/182 cluster targeting FOXO1 in mouse. Ecotoxicol Environ Saf.

[21] Joffe M (2000). Time trends in biological fertility in Britain : the lancet. Lancet..

[22] Ren Y, Li H, Wang X (2019). Family income and nutrition-related health: evidence from food consumption in China. Soc Sci Med.

[23] StataCorp. College Station TSL (2015). Stata Statistical Software: Release 14.

[24] Cooper TG, Auger J, Baker HWG, Behre HM, Haugen TB, Kruger T (2010). World Health Organization reference values for human semen characteristics. Hum Reprod Update.

[25] Barratt CL, Naeeni M, Clements S, Cooke ID (1995). Clinical value of sperm morphology for in-vivo fertility: comparison between World Health Organization criteria of 1987 and 1992. Hum Reprod.

[26] Bartoov B, Eltes F, Pansky M, Lederman H, Caspi E, Soffer Y (1993). Estimating fertility potential via semen analysis data. Hum Reprod.

[27] Crissman JW, Cooke PS, Hess RA, Marty MS, Liberacki AB (2000). Postulated human sperm count decline may involve historic elimination of juvenile iodine deficiency: a new hypothesis with experimental evidence in the rat. Toxicol Sci.

[28] Chakraborty A, Mandal J, Mondal C, Sinha S, Chandra AK (2016). Effect of excess iodine on oxidative stress markers, Steroidogenic—enzyme activities, testicular morphology, and functions in adult male rats. Biol Trace Elem Res.

[29] La Vignera S, Vita R. Thyroid dysfunction and semen quality. Int J Immunopathol Pharmacol. 2018;32:1-5. 10.1177/2058738418775241 PMC5946587 29737216

[30] Velde ET, Eijkemans R, Habbema H (2000). Variation in couple fecundity and time to pregnancy, an essential concept in human reproduction. Lancet..

[31] Hammoud A, Carrell DT, Gibson M (2010). Decreased sperm motility is associated with air pollution in Salt Lake City. Fertil Steril.

[32] Hansen C, Luben TJ, Sacks JD, Olshan A, Jeffay S, Strader L (2010). The effect of ambient air pollution on sperm quality. Environ Health Perspect.

